# The “serine code” of metabolic reprogramming: multidimensional roles of the serine synthesis pathway in tumors and novel breakthroughs for targeted therapy

**DOI:** 10.3389/fimmu.2026.1779543

**Published:** 2026-02-24

**Authors:** Peng Su, Ying Yang, Hong Zheng

**Affiliations:** 1 Department of Pathology, The Affiliated Hospital of Zunyi Medical University, Zunyi, Guizhou, China; 2 Department of Urology, The Affiliated Hospital of Zunyi Medical University, Zunyi, Guizhou, China; 3 Department of Dermatology, The Second Affiliated Hospital of Zunyi Medical University, Zunyi, Guizhou, China

**Keywords:** metabolic reprogramming, phosphoglycerate dehydrogenase (PHGDH), serine metabolism, serine synthesis pathway (SSP), targeted therapy, tumor immune microenvironment

## Abstract

As a pivotal contributor to tumor metabolism following glucose and glutamine, serine plays a crucial role in the metabolic network of tumors via its *de novo* synthesis pathway (SSP). The SSP is aberrantly activated in a variety of malignant tumors and promotes tumor progression through multi-dimensional mechanisms. On the one hand, it provides the material basis and one-carbon units required for the synthesis of nucleotides, proteins and phospholipids to support the rapid proliferation of tumor cells. On the other hand, it maintains cellular redox homeostasis by generating glutathione (GSH) and nicotinamide adenine dinucleotide phosphate (NADPH). Furthermore, it regulates the tumor immune microenvironment through metabolic reprogramming, inducing macrophage polarization and modulating T-cell function, thereby shaping an immunosuppressive microenvironment. The activity and stability of key enzymes in the SSP are precisely regulated by transcription factors (such as c-Myc, HIF-1α, and NRF2), epigenetic modifications (including m5C and m6A), and post-translational modifications (such as methylation, ubiquitination, and deacetylation). Meanwhile, the SSP forms an interactive network with tumor signaling pathways including Akt, mTOR, and EGF-ERK, collectively driving metabolic reprogramming. Therapeutic strategies targeting the SSP have emerged as a research hotspot, encompassing dietary intervention, the development of inhibitors targeting key enzymes such as phosphoglycerate dehydrogenase (PHGDH), as well as combination therapies with radiotherapy, chemotherapy and immunotherapy. Notably, these strategies have shown promising potential in reversing drug resistance to BRAF inhibitors, sorafenib, 5-fluorouracil (5-FU) and other agents, providing novel strategies for pan-cancer therapy. Through a systematic and comprehensive analysis of the multi-dimensional functions, heterogeneous regulation and roles in therapeutic resistance of the SSP across cancer types, this review aims to elucidate the conserved principles and cancer-specific characteristics of the SSP as a metabolic hub. Additionally, we discuss the prospects and unique challenges of precise intervention strategies targeting the SSP in overcoming tumor heterogeneity and drug resistance.

## 1 Introduction

Metabolic reprogramming stands as one of the hallmarks of malignant tumors, enabling tumor cells to remodel core metabolic pathways to meet the demands of rapid proliferation, invasion, metastasis, and adaptation to microenvironmental stress (1). Among the diverse metabolic networks, serine metabolism has gradually emerged as a research focus in tumor metabolism due to its multifaceted roles in energy supply, biosynthesis, and the maintenance of redox homeostasis in tumor cells. When tumor cells are subjected to metabolic stress driven by rapid proliferation, exogenous serine uptake and endogenous transformation pathways alone are insufficient to meet the biosynthetic demands, leading to the marked activation of the serine *de novo* synthesis pathway (SSP), which becomes the primary intracellular source of serine in tumor cells (2).

In recent years, with the advancement of pan-cancer studies, aberrant activation of the SSP has been successively identified across various malignant tumors, and its biological functions and regulatory mechanisms exhibit remarkable cancer-type heterogeneity (3). Although the critical role of the SSP in tumorigenesis and progression has been widely acknowledged, the complexity of its regulatory network, the molecular mechanisms underlying cancer-specific functional differences, as well as the feasibility and challenges of SSP-targeted therapy in clinical translation, remain to be thoroughly elucidated. Therefore, a systematic review of the functions, regulatory mechanisms, and targeted therapeutic strategies of the SSP in pan-cancer will not only provide a theoretical basis for understanding the conserved and distinctive features of tumor metabolic reprogramming, but also offer novel insights into the development of pan-cancer precision therapeutic regimens targeting the SSP.

Against this background, this review begins with the metabolic mechanism of the SSP, and elaborates in detail on its core functions in pan-cancer, multi-level regulatory networks, as well as current therapeutic strategies and challenges targeting the SSP, aiming to provide a reference for subsequent tumor metabolism research and clinical translation.

## 2 Overview of serine metabolism

Serine is a non-essential amino acid that participates in a wide spectrum of cellular processes, including protein synthesis, cell proliferation and metabolic homeostasis (4). It ranks as the third major contributor to tumor metabolism, following glucose and glutamine (5). Serine can be taken up by cells via multiple membrane transporters, and can also be synthesized *de novo* intracellularly. Although serine can be derived from the degradation of cellular proteins and the interconversion with glycine, these pathways alone are insufficient to meet the metabolic demands of biological tissues, especially in pathological conditions (6). The *de novo* serine synthesis pathway (SSP) substantially enhances the intracellular availability of serine (7).

The *de novo* serine synthesis pathway, also referred to as the serine biosynthetic pathway, is a branch pathway of glycolysis. Initiating with the glycolytic intermediate 3-phosphoglycerate, this pathway proceeds through three sequential enzymatic reactions. First, 3-phosphoglycerate is oxidized to 3-phosphohydroxypyruvate with concomitant production of NADH, catalyzed by phosphoglycerate dehydrogenase (PHGDH). Subsequently, 3-phosphohydroxypyruvate accepts an amino group from α‑ketoglutarate under the catalysis of phosphoserine aminotransferase 1 (PSAT1), yielding phosphoserine. Finally, phosphoserine is hydrolyzed to serine by phosphoserine phosphatase (PSPH) (7). In the cytoplasm, serine can be converted to glycine by serine hydroxymethyltransferase 1 (SHMT1), whereas this conversion is mediated by serine hydroxymethyltransferase 2 (SHMT2) in mitochondria (8). During this metabolic reaction, the one-carbon unit cleaved from serine is transferred to tetrahydrofolate (THF) to form 5,10-methylenetetrahydrofolate (CH2-THF). CH2-THF is then catalyzed by 5,10-methylenetetrahydrofolate reductase (MTHFR) to generate 5-methyltetrahydrofolate (5-CH3-THF), which further donates its methyl group to regenerate THF, thereby completing the folate cycle (9).

In recent years, accumulating studies have demonstrated that the *de novo* serine synthesis pathway is aberrantly activated in a variety of malignancies. SSP supports the anabolic processes required for the rapid proliferation of cancer cells. Conversely, serine deprivation triggers cellular stress and adaptive metabolic remodeling, and subsequently suppresses tumor progression (10). Accordingly, the *de novo* serine synthesis pathway has emerged as a central focus in the field of cancer metabolism research (4–12).

## 3 Core roles of the *de novo* serine synthesis pathway in malignant tumors

**Figure 1:**
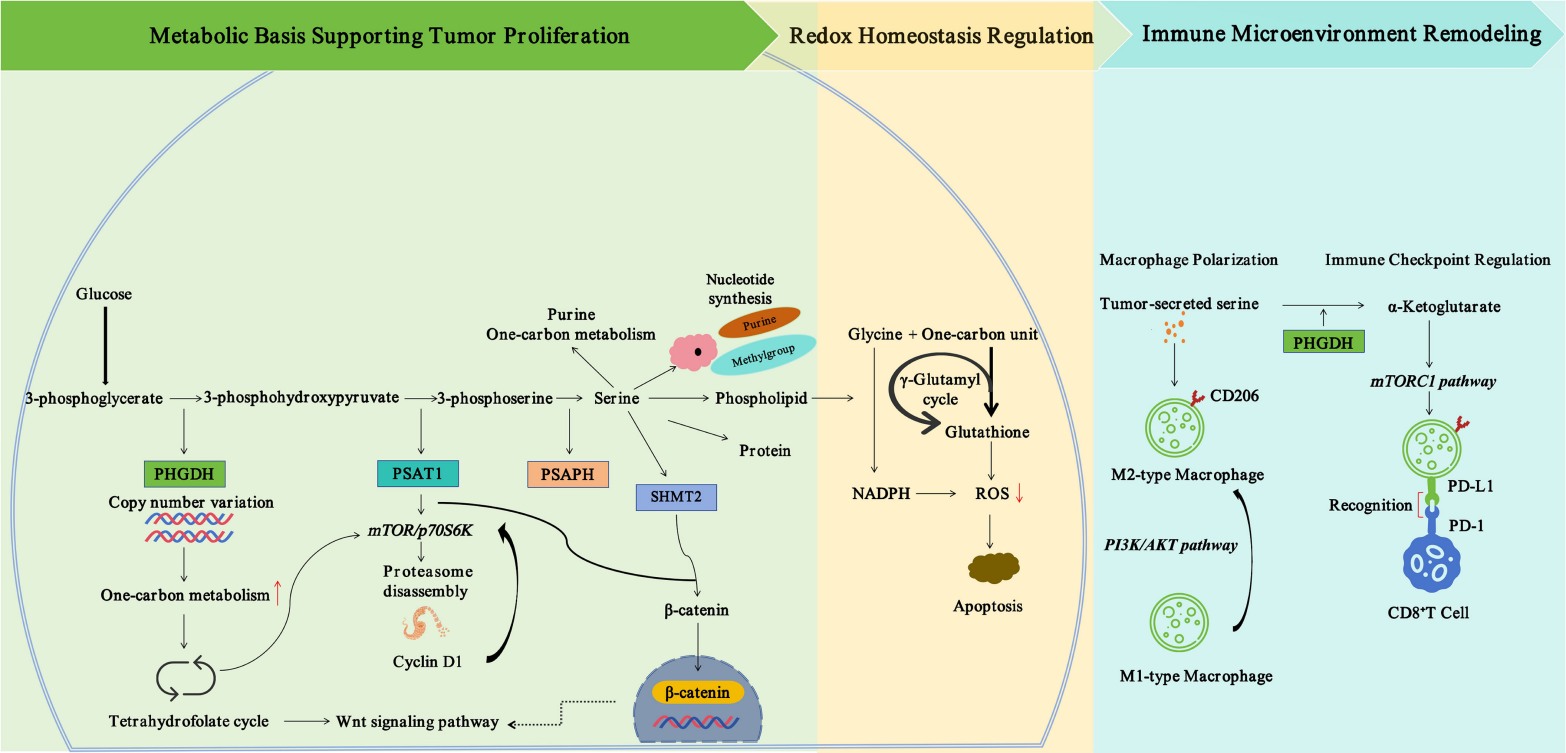
Core roles of the *de novo* serine synthesis pathway in tumors. This figure illustrates the core mechanisms by which the *de novo* serine synthesis pathway (SSP) drives malignant tumor progression from three dimensions: the metabolic basis for tumor proliferation, redox homeostasis regulation, and tumor immune microenvironment remodeling. 1. Metabolic basis for tumor proliferation: The glycolytic intermediate 3-phosphoglycerate is oxidized to 3-phosphohydroxypyruvate under the catalysis of phosphoglycerate dehydrogenase (PHGDH). Subsequently, 3-phosphohydroxypyruvate is converted to 3-phosphoserine by phosphoserine aminotransferase 1 (PSAT1), and finally hydrolyzed to serine via phosphoserine phosphatase (PSPH). This process provides the material foundation and one-carbon units required for the biosynthesis of nucleotides, proteins and phospholipids to support the rapid proliferation of tumor cells. PSAT1 regulates the degradation of cyclin D1 through the mTOR/p70S6K signaling axis, further promoting cell proliferation. In addition, serine hydroxymethyltransferase 2 (SHMT2) forms a positive feedback loop with β-catenin to activate the Wnt signaling pathway, thereby driving tumor progression. 2. Regulation of redox homeostasis: NADPH generated via serine metabolism can scavenge intracellular reactive oxygen species (ROS) and maintain redox homeostasis in tumor cells. When the SSP is inhibited, the production of NADPH decreases and ROS accumulates, ultimately inducing apoptosis in tumor cells. 3. Remodeling of the tumor immune microenvironment: PHGDH-mediated *de novo* serine synthesis promotes the production of α-ketoglutarate, which activates the mTORC1 signaling pathway in macrophages, induces their polarization toward the M2 phenotype and sustains high expression of programmed death ligand 1 (PD-L1). Consequently, the cytotoxic function of CD8^+^ T cells is suppressed.

The serine synthesis pathway exerts multiple critical functions in cancer cells. First, it directly supplies precursors for the biosynthesis of proteins, nucleotides, and phospholipids. Second, the intermediate metabolites of this pathway serve as vital sources for the generation of glycine and one-carbon units. The latter directly participate in purine synthesis and sustain intracellular methylation cycles, which are indispensable for DNA and histone methylation. Finally, this pathway also modulates the tumor immune microenvironment through metabolic reprogramming, as illustrated in **Figure 1**.

### 3.1 Providing the material basis for tumor proliferation

Serine is catalyzed by serine hydroxymethyltransferase (SHMT) to generate 5,10-methylenetetrahydrofolate with tetrahydrofolate as the carbon carrier, providing essential one-carbon units for the biosynthesis of nucleotides, amino acids and other macromolecules, thereby participating in the regulation of cell proliferation and metabolism (9, 13). A one-carbon unit refers to a functional group containing a single carbon atom produced during the catabolism of certain amino acids. As key precursors for purine and pyrimidine synthesis, one-carbon units act as a critical bridge connecting amino acid metabolism and nucleotide biosynthesis (13, 14). The reversible interconversion between serine and glycine represents the primary route through which one-carbon metabolism supplies one-carbon units to the folate cycle (15, 16).

This reaction contributes to cysteine biosynthesis via the transsulfuration pathway, which is also metabolically linked to the homocysteine-methionine cycle (16, 17). Therefore, serine functions as a pivotal precursor for the synthesis of nucleotides, proteins and phospholipids. Activation of its *de novo* synthesis pathway directly replenishes metabolic substrates for rapidly proliferating tumor cells (18). For instance, gene amplification of PHGDH in melanoma, breast cancer and non-small cell lung cancer leads to a remarkable elevation in enzymatic activity and increased serine production. Through one-carbon metabolism, this process provides methyl donors for DNA replication and accelerates the cell cycle progression of tumor cells (3, 19).

In colorectal cancer, phosphoserine aminotransferase 1 (PSAT1) regulates the degradation of cyclin D1 via the mTOR/p70S6K signaling axis, further promoting cell proliferation (20, 21). In contrast, serine hydroxymethyltransferase 2 (SHMT2) forms a positive feedback loop with β-catenin to activate the Wnt signaling pathway, acting as a crucial driver of colorectal cancer progression (22).

### 3.2 Maintaining redox homeostasis in tumor cells

The *de novo* serine synthesis pathway eliminates intracellular reactive oxygen species (ROS) in tumor cells and prevents oxidative stress-induced apoptosis by promoting the production of glutathione (GSH) and nicotinamide adenine dinucleotide phosphate (NADPH) (22, 23). Glutathione is a tripeptide composed of glutamate, cysteine and glycine. The active sulfhydryl group (-SH) in its molecular structure readily undergoes oxidative dehydrogenation, making GSH the major physiological free radical scavenger in organisms (24, 25). GSH can efficiently neutralize excessive intracellular ROS, as well as endogenous and exogenous electrophilic compounds, thereby maintaining intracellular redox homeostasis and alleviating cellular damage caused by oxidative stress (26, 27).

Nicotinamide adenine dinucleotide (NAD^+^) and its reduced form (NADH) constitute a pivotal intracellular redox couple. Acting as enzyme cofactors, they mediate hydrogen atom transfer in catabolic redox reactions (26). Besides functioning as a coenzyme in redox reactions, NAD^+^ can be phosphorylated to nicotinamide adenine dinucleotide phosphate (NADP^+^) under the catalysis of nicotinamide adenine dinucleotide kinase (NADK). NADP^+^ serves as a hydride acceptor to generate NADPH, which is extensively involved in cellular anabolic processes and the antioxidant defense system (28). Therefore, the *de novo* serine synthesis pathway is indispensable for maintaining cellular redox homeostasis and regulating cellular energy metabolism.

In hepatocellular carcinoma (HCC), activation of PHGDH promotes the synthesis of GSH and NADPH, markedly reducing intracellular ROS levels. Conversely, PHGDH knockdown or inhibition of its enzymatic activity leads to ROS accumulation and induces oxidative stress-mediated death in HCC cells (29, 30). Another study demonstrated that FBXO7 directly interacts with PRMT1, resulting in decreased PRMT1 protein abundance and suppressed methylation of PHGDH. This consequently impairs serine synthesis, triggers ROS accumulation, and ultimately inhibits the growth of HCC cells (31). Recent research has revealed that hypoxia upregulates PHGDH expression in human colorectal cancer (CRC) cell lines, and PHGDH inhibition elevates ROS levels, thereby enhancing radiosensitivity (32). In ovarian cancer, treatment with the PHGDH inhibitor CBR-5884 increases intracellular ROS levels by activating the ROS/Wnt/β-atenin signaling axis, suppressing the proliferation, migration and invasion of ovarian cancer cells (33). In prostate cancer, PHGDH contributes to the resistance of tumor cells against enzalutamide-induced cytotoxicity by sustaining redox balance, representing a key mechanism underlying drug resistance (34).

### 3.3 Shaping the immunosuppressive tumor microenvironment

Metabolic reprogramming represents one of the core hallmarks of malignant tumors. It not only provides the material and energetic basis for the unlimited proliferation of tumor cells, but also reshapes the tumor immune microenvironment (TIME) by regulating metabolite levels (including lactate, glutamate, serine, etc.) and mediating epigenetic modifications such as histone lactylation and methylation. These processes exert inhibitory or tumor-promoting effects on the activation, proliferation, differentiation and function of immune cells, forming a critical bridge linking tumor cell intrinsic metabolism and tumor immunity (35, 36). Aberrant activation of the *de novo* serine synthesis pathway modulates the tumor immune microenvironment through metabolic reprogramming (37, 38).

The balance between tumorigenesis and antitumor immune function governed by serine metabolism is determined by the abundance and activation status of distinct cell populations, as well as the expression profiles of various immune mediators and regulatory factors within the tumor microenvironment (TME) (37). As a key oncogenic metabolite, serine participates in the generation, recruitment and functional regulation of immune cells (39). Serine acts as an essential regulator of macrophage polarization. Genetic deletion or pharmacological inhibition of serine synthesis pathway-related enzymes drives the polarization of tumor-associated macrophages (TAMs) from the M2 to the M1 phenotype (40, 41).

Bladder cancer cells with PSAT1 overexpression can secrete serine, which activates the PI3K/Akt pathway in macrophages and induces their polarization toward the M2 phenotype, thereby suppressing antitumor immune responses (42). PHGDH-mediated *de novo* serine synthesis promotes the production of α-ketoglutarate, which activates the mTORC1 signaling pathway in macrophages, facilitates the acquisition of the M2 macrophage phenotype and sustains high PD-L1 expression, ultimately inhibiting the cytotoxic function of CD8^+^ T cells. In contrast, PHGDH knockout attenuates tumor growth, reduces TAM infiltration, promotes the phenotypic conversion of M2-like TAMs to an M1-like state, downregulates PD-L1 expression, and consequently enhances antitumor T-cell immunity (43).

Serine generates multiple pivotal metabolites through the one-carbon metabolic pathway (SGP), regulating the development, proliferation and differentiation of CD8^+^ T cells, effector T cells and regulatory T cells (Tregs). Dysregulation of the *de novo* serine synthesis pathway impairs T-cell proliferation and their antitumor immune function (39). Hypermethylation of PSAT1 is associated with T-cell dysfunction, shortened survival and impaired immune cell infiltration in breast cancer (44). Furthermore, PSAT1 expression in lung adenocarcinoma shows a significantly positive correlation with tumor mutational burden (TMB), but a negative correlation with tumor immune dysfunction and exclusion (TIDE) (44). In non-small cell lung cancer (NSCLC), PSAT1 overexpression is correlated with poor prognosis and aberrant immune cell infiltration (45). SHMT2 expression is markedly associated with CD8^+^ T-cell infiltration and is highly upregulated in breast cancer and papillary renal cell carcinoma (46).

In addition, activation of the serine synthesis pathway in glioblastoma attenuates the therapeutic efficacy of CAR-T cells, whereas PHGDH inhibition restores the antitumor activity of immune cells by enhancing STAT1 signaling (47).

Taken together, the *de novo* serine synthesis pathway (SSP) serves as a critical component of tumor metabolic reprogramming, driving malignant tumor progression through multi-dimensional regulatory mechanisms. Its core functions are manifested in three major aspects: material supply, maintenance of intracellular homeostasis, and immune regulation, which collectively form a tumor-promoting synergistic effect. At the proliferative level, the SSP provides essential biosynthetic support for tumor cells by activating enzymes including PHGDH and PSAT1. Serine participates in one-carbon metabolism via serine hydroxymethyltransferases (SHMTs), supplying synthetic precursors such as nucleotides. Meanwhile, the SSP modulates the mTOR/p70S6K and Wnt signaling pathways to accelerate cell cycle progression, exerting pro-proliferative effects in a variety of malignancies including melanoma and breast cancer. In terms of redox homeostasis, the SSP eliminates reactive oxygen species (ROS) through the production of glutathione (GSH) and nicotinamide adenine dinucleotide phosphate (NADPH), thereby preventing tumor cell apoptosis and mediating drug resistance. PHGDH acts as a central regulator, and its inhibitors can disrupt redox balance, offering promising therapeutic targets for tumor intervention.

In the context of tumor immune microenvironment remodeling, the SSP induces an immunosuppressive phenotype, promotes M2-type macrophage polarization, sustains high PD-L1 expression, impairs the cytotoxic function of CD8^+^ T cells, and compromises the therapeutic efficacy of CAR-T cells. In contrast, inhibition of PHGDH can reverse the immunosuppressive state. Therefore, we conclude that the SSP establishes the fundamental metabolic basis for malignant tumor progression. Its key enzymes and the entire pathway not only act as driving factors of tumor development, but also provide theoretical support and potential targets for targeted therapy and its combination with immunotherapy.

## 4 Research progress of the serine synthesis pathway in pan-cancer

**Figure 2:**
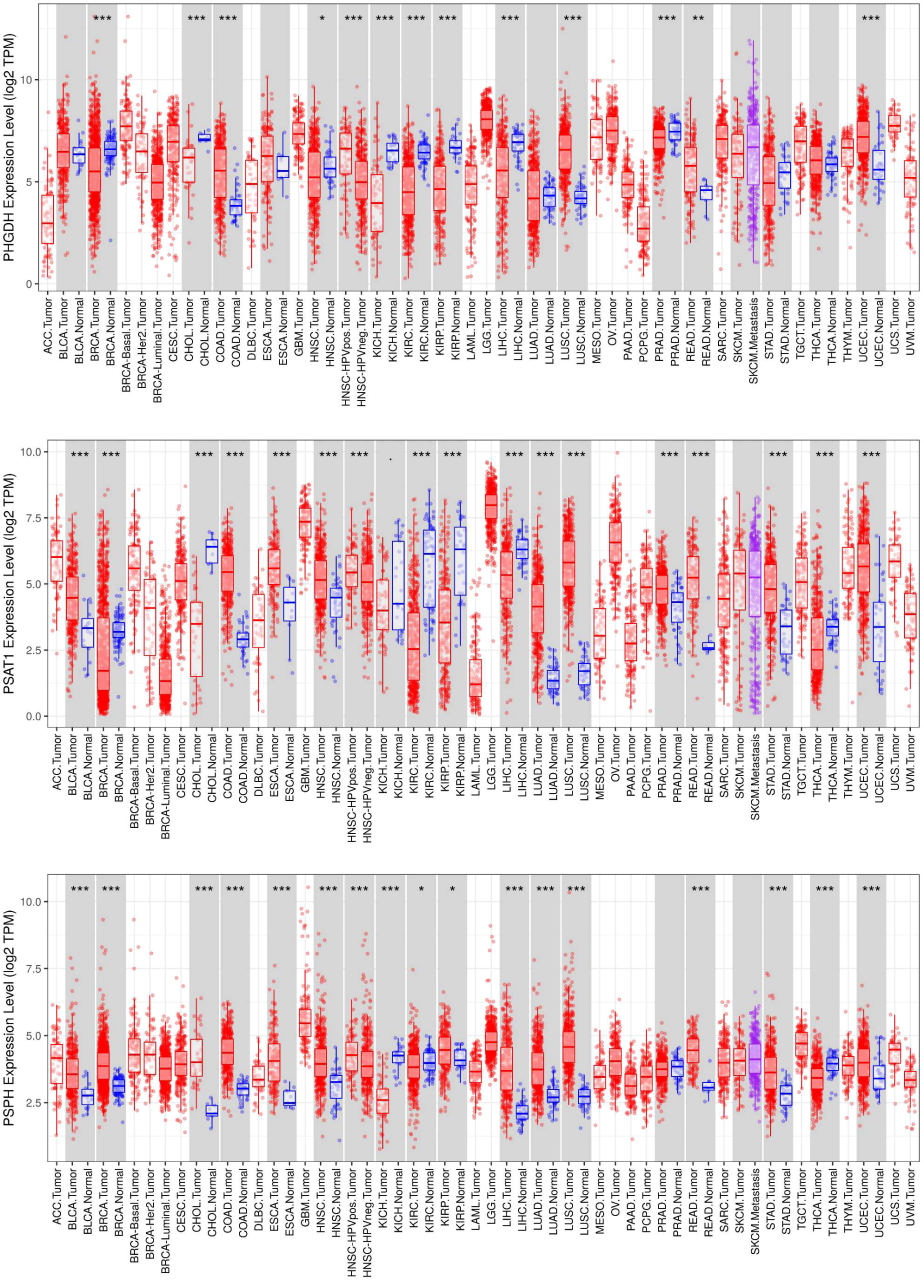
Expression of enzymes associated with the serine synthesis pathway across pan-cancer. The differential transcriptional expression of key enzymes involved in the *de novo* serine synthesis pathway (including PHGDH, PSAT1 and PSPH) was systematically analyzed across pan-cancer specimens using the TIMER online database. *P < 0.05; **P < 0.01; ***P < 0.001.

The transcriptional expression differences of key enzymes in the *de novo* serine synthesis pathway (including PHGDH, PSAT1 and PSPH) across pan-cancer specimens were systematically analyzed using the Timer online database (**Figure 2**), revealing the expression heterogeneity and potential tumor-specific expression patterns of these enzymes in distinct cancer types. In recent years, with the advancement of pan-cancer studies, aberrant activation of the SSP has been successively identified in various malignant tumors. Although SSP activation represents a common characteristic across multiple cancers, its driving forces, key regulatory nodes and functional outcomes exhibit considerable cancer-type specificity. The following sections elaborate on these aspects in detail for individual cancer types, and this chapter concludes with a comprehensive analysis.

### 4.1 High expression of all three key SSP enzymes: PHGDH, PSAT1 and PSPH

#### 4.1.1 Non-small cell lung cancer

In non-small cell lung cancer (NSCLC), the expression of PHGDH, the rate-limiting enzyme of the SSP, is significantly higher in tumor tissues than in adjacent normal tissues, and is positively correlated with TNM stage. Patients with high PHGDH expression exhibit markedly shorter overall survival (3, 48). Wild-type IDH1 (IDH1^WT) can function as a non-enzymatic scaffold protein to interact with PHGDH, a key enzyme in the *de novo* serine synthesis pathway, and the RNA-binding protein FXR1. This interaction prevents their binding to Parkin, an E3 ubiquitin ligase, thereby maintaining the protein stability of both PHGDH and FXR1 (49).

Accumulation of FXR1 further enhances the stability of PSAT1 mRNA and promotes its translation, ultimately upregulating the expression of PHGDH and PSAT1 and activating the *de novo* serine synthesis pathway. This leads to an imbalance in the glutathione/reactive oxygen species (ROS) axis and enhanced pyrimidine biosynthesis, which in turn sustains the cancer stem cell-like properties of lung cancer cells and drives the malignant progression of NSCLC (49). In NSCLC, NRF2 upregulates the expression of PHGDH, PSAT1 and SHMT2 by activating ATF4, thereby driving SSP activation. High expression of these genes is closely associated with poor prognosis in patients with NSCLC (50).

Metabolites involved in the serine/glycine pathway are markedly depleted in the plasma of NSCLC patients after radiotherapy and in corresponding cellular models, indicating that tumor cells utilize these metabolites to facilitate DNA damage repair and cell survival. Correspondingly, combined treatment with sertraline, an inhibitor of serine/glycine conversion, and radiotherapy significantly impairs the proliferation, clonogenicity and stem cell self-renewal capacity of NSCLC cells, and also effectively suppresses tumor growth in *in vivo* experiments (9).

#### 4.1.2 Colorectal cancer

Genes involved in the SSP are generally highly expressed in colorectal cancer and are correlated with unfavorable patient prognosis (2, 32). Serine hydroxymethyltransferase 2 (SHMT2), a pivotal enzyme linking the SSP and one-carbon metabolism, specifically interacts with β-catenin in colorectal cancer, leading to sustained activation of the Wnt signaling pathway. SHMT2 promotes the nuclear localization of β-catenin and facilitates its binding to the TCF/LEF transcription factor complex, thereby upregulating the expression of downstream target genes including cyclin D1 and c-Myc, and accelerating the proliferation and invasion of colorectal cancer cells.

Meanwhile, activation of the Wnt pathway in turn promotes the transcription of SHMT2, forming a positive feedback loop of the SHMT2–Wnt/β-catenin axis that further strengthens the metabolic output of the SSP (22). Furthermore, under low-glucose conditions, SIRT3-mediated deacetylation of SHMT2 stabilizes its protein structure, enabling colorectal cancer cells to maintain SSP activity even in the nutrient-deficient tumor microenvironment (51). Colorectal cancer cells with defective oxidative phosphorylation (OXPHOS) upregulate the expression of PHGDH and PSAT1 to increase the synthesis of serine and glutathione, scavenge intracellular ROS and maintain redox homeostasis, thus supporting cell survival and proliferation under restricted energy metabolism.

Clinical samples have confirmed that approximately 82% of OXPHOS-deficient colorectal cancer tissues harbor mtDNA mutations, and the SSP activity in these tumors is significantly higher than that in normal tissues (52). Metabolic plasticity of serine metabolism represents one of the key pathways underlying 5-FU resistance in colorectal cancer. Enhanced SSP activity promotes colorectal cancer growth and contributes to resistance to 5-FU (53). Reprogramming of mitochondrial serine metabolism in colorectal cancer facilitates purine nucleotide biosynthesis and enables drug-resistant cells to prevent the accumulation of drug-induced DNA damage, thereby promoting resistance to 5-FU (54).

The transcription factor FOXC1 has also been shown to reprogram serine metabolism under serine-deprived conditions to promote colorectal cancer proliferation and 5-fluorouracil resistance. In contrast, serine deprivation or SSP blockade can restore the sensitivity of colorectal cancer cells to 5-FU (55). In addition, the mechanism by which PSAT1 induces cyclin D1 degradation via the mTOR/p70S6K pathway has been verified to be associated with chemoresistance in colorectal cancer (21), and its expression level is negatively correlated with the therapeutic response to oxaliplatin in patients (56).

#### 4.1.3 Endometrial cancer

PHGDH, PSAT1 and PSPH are significantly upregulated in uterine corpus endometrial carcinoma (UCEC) compared with normal tissues (57). High PHGDH expression is significantly associated with poor prognosis in endometrial cancer. Tissue immunohistochemistry has confirmed that high-grade endometrial cancer exhibits higher PHGDH IHC scores, and patients with high scores present increased recurrence rates and shortened survival (58). Serine metabolism is abnormally active in endometrial cancer. A large-scale prospective analysis has revealed that higher circulating serine levels are associated with a lower risk of endometrial cancer, suggesting that systemic serine metabolic status may serve as a risk indicator for disease development (59).

However, the situation is completely opposite within tumor tissues: cancer cells activate endogenous serine synthesis to meet the demands of rapid growth. ¹H HR-MAS NMR metabolomics has demonstrated that serine levels are significantly elevated in endometrial cancer tissues (G1, G2, G3) compared with normal endometrial tissues, and increase with tumor grade, showing a positive correlation with malignancy (60).

#### 4.1.4 Hepatocellular carcinoma

PRMT1 binds to valine 83 (V83) of PHGDH and catalyzes the methylation of arginine 236 (R236) on PHGDH, thereby increasing the affinity of PHGDH for its substrate 3-phosphoglycerate and markedly enhancing enzymatic activity. This modification not only promotes the *de novo* synthesis of serine and glycine, but also alleviates oxidative stress by increasing the production of GSH and NADPH to eliminate intracellular ROS, thus promoting the survival and proliferation of hepatocellular carcinoma cells *in vitro* and *in vivo* (29).

Another study has found that overexpression of FBXO7 inhibits the SSP by degrading PRMT1, inducing oxidative stress and suppressing tumor growth in hepatocellular carcinoma. In contrast, FBXO7 knockdown significantly increases the synthesis of serine and glycine and promotes the proliferation of hepatocellular carcinoma xenografts (31). m6A modification plays a critical role in hepatocellular carcinoma progression and acquired resistance to sorafenib and lenvatinib by stabilizing the mRNA of SSP-related genes (61). Treatment with m6A inhibitors effectively suppresses the SSP, induces oxidative stress, and resensitizes hepatocellular carcinoma cells to targeted therapies (61).

Hyperactivation of the SSP increases the supply of substrates for m6A methylation, further sustaining the high expression of PCK2 and NRF2, ultimately resulting in significantly reduced sensitivity of hepatocellular carcinoma cells to lenvatinib (62).

#### 4.1.5 Glioblastoma

In glioblastoma (GBM), especially within the serine/glycine (S/G)-deprived brain microenvironment, the SSP is significantly activated (63). Transcriptomic and metabolomic analyses have shown that PHGDH, PSAT1 and PSPH are highly expressed in GBM cells, and their expression levels are closely associated with poor patient prognosis (64–66). For instance, in clinical specimens, PHGDH expression is remarkably upregulated in glioma stem cells (GSCs). MYC mediates PHGDH activation, which enhances GSC self-renewal by regulating redox homeostasis, promoting on-carbon metabolism and facilitating the DNA damage response via SSP activation, thereby driving GSC malignant progression and radioresistance in GBM (64). In addition, the enrichment of the SSP is positively correlated with the expression of tumor stemness markers such as CD133, further promoting tumor malignant progression (67).

In terms of expression synergy, the concurrent high expression of all three key enzymes indicates that the SSP is fully activated in these cancer types. By enhancing *de novo* serine synthesis throughout the entire pathway, tumor cells meet the demands of proliferation, redox homeostasis maintenance and adaptation to the microenvironment. Full pathway activation may represent the core metabolic strategy employed by tumor cells to cope with environmental stress and sustain malignant phenotypes.

### 4.2 Selective high expression of one or two enzymes

#### 4.2.1 Breast cancer

PHGDH is highly expressed in approximately 70% of estrogen receptor (ER)-negative breast cancers. Studies have demonstrated that PHGDH overexpression enables breast cancer cells to redirect more glutamine into the tricarboxylic acid cycle via the SSP, thereby supporting rapid cell proliferation (68, 69). Moreover, PHGDH protein levels are positively correlated with recurrence in triple-negative breast cancer (TNBC), and its high expression in TNBC patient tissues is closely associated with early metastasis and relapse. The PHGDH inhibitors NCT-503 and CBR-5884 significantly suppress cell proliferation, with more potent effects under serine/glycine-deprived conditions (70).

In addition, a synthetic lethal relationship exists between the oncogenic activity of IDH2 and the serine synthesis pathway in both TNBC and HER2-positive breast cancer, rendering cells with high IDH2 expression particularly sensitive to PHGDH or PSAT1 inhibition (71). Notably, the epithelial marker E-cadherin has also been found to promote breast cancer progression and metastasis by upregulating the SSP, and PHGDH inhibition selectively impedes the proliferation and metastatic potential of E-cadherin-positive cancer cells (72). Hypermethylation of PSAT1 is associated with T-cell dysfunction, shortened survival and impaired immune cell infiltration in breast cancer (44).

#### 4.2.2 Pancreatic cancer

The tumor microenvironment of pancreatic cancer is nutrient-deficient, especially with regard to serine limitation. Serine deprivation specifically reduces the mRNA translation efficiency of serine codons TCC and TCT, leading to the selective translation and secretion of nerve growth factor (NGF) by pancreatic cancer cells. NGF promotes tumor innervation, enabling nerve axons to release serine and feed back to tumor cells (73).

In pancreatic cancer, the accumulation of 3-phosphoglycerate (3-PG) induces PHGDH expression and facilitates serine biosynthesis, thereby driving tumor growth. Research has also shown that under serine-deficient conditions, pancreatic ductal adenocarcinoma (PDAC) cells maintain intracellular serine levels by upregulating PHGDH, adapting to the harsh microenvironment and promoting tumor progression (74). Activating mutations in the Kras gene represent one of the core drivers of PDAC. Activated KRAS upregulates the expression of key SSP enzymes including PHGDH, promotes *de novo* serine synthesis, and reduces the dependence of tumor cells on environmental serine (75). As the rate-limiting enzyme of the SSP, PHGDH high expression is closely correlated with the malignancy of PDAC, and targeting PHGDH effectively inhibits the growth of pancreatic cancer cells under nutrient-deprived conditions (3, 74, 76).

#### 4.2.3 Bladder cancer

Frequent FGFR3 mutations in bladder cancer constitute one of the core drivers of SSP activation. Activated mutant FGFR3 (aFGFR3) upregulates the expression of key SSP enzymes such as PHGDH and PSAT1 through downstream signaling pathways, promoting intracellular *de novo* serine synthesis in bladder cancer cells (42). This process not only supports rapid tumor cell proliferation but also fosters immunosuppression by remodeling the tumor microenvironment.

Specifically, elevated serine synthesis subsequently activates the PI3K/Akt pathway in tumor-associated macrophages, shifting macrophages toward an immune-inert phenotype. Such macrophages exhibit markedly impaired T-cell recruitment and antigen-presenting capacity, establishing an “immune-desert” tumor microenvironment that suppresses antitumor immune responses and facilitates immune escape in bladder cancer (42). Serine hydroxymethyltransferase (SHMT) is a pivotal enzyme in serine metabolism, especially in one-carbon metabolism. SHMT2 expression is significantly upregulated in bladder cancer and is closely associated with poor patient prognosis. SHMT2 modulates the growth, migration and apoptosis of bladder cancer cells by regulating the expression levels of E-cadherin and N-cadherin, thus promoting malignant biological behaviors (77).

Furthermore, loss of amylo-1,6-glucosidase (AGL), a glycogen debranching enzyme, leads to elevated SHMT2 levels, which in turn increases glycine and purine nucleotide synthesis and further supports tumor cell proliferation (78). PHGDH expression is significantly higher in high-grade bladder cancer than in low-grade tumors, and patients with high PHGDH expression exhibit shorter survival than those with low expression. In bladder cancer cell lines, PHGDH knockdown markedly suppresses proliferative capacity and induces apoptosis. Hypomethylation of the PHGDH promoter region represents a crucial epigenetic mechanism underlying its high expression in bladder cancer. Hypomethylation drives persistent SSP activation, supporting tumor proliferation and chemoresistance (79).

#### 4.2.4 Prostate cancer

The transcription factor NKX2–1 is significantly overexpressed in neuroendocrine prostate cancer (NEPC), directly binding to the promoters of PHGDH and PSAT1 to transcriptionally activate SSP gene expression. Cells overexpressing NKX2–1 can proliferate in serine/glycine-free medium, whereas NKX2–1 knockdown inhibits invasive capacity (80).

Studies have revealed that in the most aggressive subtype of castration-resistant prostate cancer (CRPC), downregulation of protein kinase C λ/ι (PKCλ/ι) upregulates *de novo* serine synthesis via the mTORC1/ATF4 signaling pathway. This metabolic reprogramming not only supports cell proliferation but also elevates intracellular S-adenosylmethionine (SAM) levels, promoting epigenetic alterations and ultimately inducing the acquisition of NEPC characteristics (81, 82). In addition, changes in serine levels show potential in distinguishing low-grade (Gleason score 6) from high-grade (Gleason score 7) prostate cancer, with more pronounced abnormalities in serine metabolism observed in high-grade tumors (83, 84).

PLK1 is highly expressed in high-grade prostate cancer and induces PHGDH degradation by phosphorylating residues Ser512, Ser513 and Ser517. This forces cancer cells to rely on exogenous serine uptake, which is preferentially channeled into sphingolipid biosynthesis to facilitate metastatic colonization (85). Phospholipase Cϵ (PLCϵ) regulates serine/glycine metabolism by modulating the dephosphorylation and nuclear translocation of Yes-associated protein (YAP). Knockdown of PLCϵ or treatment with verteporfin, a specific YAP inhibitor, effectively suppresses serine/glycine secretion and the growth of prostate cancer cells (86).

#### 4.2.5 Melanoma

In melanoma, the gene copy number of PHGDH is significantly increased. This molecular alteration ensures tumor cell survival and proliferation in the microenvironment with low physiological serine concentrations. Further studies have confirmed that both dietary serine supplementation and genetic PHGDH overexpression effectively drive malignant progression of melanoma by elevating intracellular serine levels (87).

Evidence also suggests that PHGDH regulates the brain metastasis of melanoma. Melanoma metastases are highly dependent on the SSP in nutrient-limited environments, and PHGDH knockout or inhibition markedly suppresses the metastatic capacity of melanoma in mouse models (88). Moreover, PHGDH upregulation confers resistance to MEK inhibitors in NRAS-mutant melanoma, whereas targeted PHGDH inhibition restores the sensitivity of resistant tumors to MAPK signaling pathway inhibitors by reducing intracellular glutathione levels and enhancing oxidative stress (89, 90).

Emerging evidence indicates that serine metabolism may also influence the efficacy of immunotherapy. Analysis of data from melanoma patients receiving anti-PD-1 therapy reveals that the expression of serine metabolic enzymes, including PHGDH, PSPH, PSAT1, SHMT1 and SHMT2, is correlated with therapeutic response and immune scores. Notably, reducing environmental L-serine levels has been shown to enhance natural killer (NK) cell function, thereby improving the efficacy of PD-1 immunotherapy (91).

#### 4.2.6 Lung adenocarcinoma

PHGDH expression is particularly prominent in lung adenocarcinoma, closely associated with enhanced cell proliferation and migration, and maintains redox homeostasis by regulating glutathione (GSH) and pyrimidine synthesis (3). In lung adenocarcinoma, PSAT1 inhibits mTORC1 activation and enhances basal autophagy levels by binding to GTP-bound RagB GTPase. Increased SSP flux mediated by PSAT1 provides glycine for GSH synthesis, reducing ROS levels by approximately 40%, protecting cancer cells from oxidative damage and ultimately promoting tumor growth (92).

#### 4.2.7 Acute myeloid leukemia

Acute myeloid leukemia (AML) cells, especially leukemia stem cells, are highly dependent on the SSP maintained by the m6A-IGF2BP3 axis. IGF2BP3 knockdown, degradation, and serine/glycine deprivation exert synergistic inhibitory effects on AML *in vitro* and *in vivo* (93).

Studies have also found that AML cells, particularly the subtype harboring FLT3-ITD mutations, exhibit high serine dependence. When exogenous glutamine is depleted, AML cells significantly upregulate the expression of key SSP enzymes such as PHGDH, compensating for metabolic deficits by enhancing *de novo* serine synthesis to sustain cell survival and proliferation (94). In a fructose-rich environment, AML cells become more reliant on the SSP. Increased SSP flux facilitates the production of α-ketoglutarate from glutamine, supporting proliferation under glucose-deficient conditions (95).

Research has identified that N-acetyltransferase 10 (NAT10)-mediated RNA acetylation (ac4C) promotes the uptake of exogenous serine by AML cells through enhancing the translation of the serine transporter SLC1A4. Concurrently, it activates the HOXA9/MENIN pathway to upregulate the expression of key enzymes in the serine synthesis pathway and boost endogenous serine production, thereby remodeling serine metabolism and driving leukemogenesis (96). As the rate-limiting enzyme of the SSP, PHGDH high expression is closely correlated with the malignancy of AML (97). Animal experiments have confirmed that PHGDH knockdown or treatment with its specific inhibitors effectively suppresses the growth of AML xenografts without significant toxicity to normal hematopoietic stem cells (95).

The expression patterns characterized by high expression of only one or two enzymes in these cancer types may stem from the unique demand for carbon source diversion from upstream glycolysis in corresponding tumors. Meanwhile, the activities of PSAT1 and PSPH could be regulated by non-transcriptional mechanisms, such as post-translational modifications. It is also possible that the serine requirement of these malignancies can be partially met through exogenous serine uptake.

### 4.3 Conserved mechanisms of the serine synthesis pathway (SSP) and cancer-type-specific adaptive strategies across pan-cancer

Based on the integrated analysis of the aforementioned 12 cancer types, we identified that the SSP exhibits both conserved regulatory mechanisms and cancer-type-specific regulatory characteristics across pan-cancer. This regulatory pattern provides critical support for tumor metabolic classification and precision therapy. The conserved mechanisms of the SSP, including redox homeostasis, one-carbon metabolism, and drug resistance regulation, can serve as common targets for cross-cancer therapeutic interventions. In contrast, cancer-type-specific adaptive strategies, such as driver mutation dependence, microenvironmental stress adaptation, and epigenetic dysregulation, necessitate the development of tailored therapeutic regimens.

#### 4.3.1 Conserved regulatory mechanisms across cancer types

The conserved functions of the SSP across pan-cancer are predominantly concentrated in three core domains: the maintenance of redox homeostasis, the support of one-carbon metabolism, and the regulation of tumor drug resistance. Regarding redox homeostasis regulation, most cancer types utilize glycine produced via the SSP to synthesize glutathione (GSH), which scavenges reactive oxygen species (ROS) to alleviate oxidative stress. For instance, high expression of phosphoglycerate dehydrogenase (PHGDH) has been validated to protect cancer cells from oxidative damage by elevating GSH levels in lung cancer, hepatocellular carcinoma, and melanoma. In terms of one-carbon metabolism support, intermediate metabolites of the SSP participate in one-carbon unit transfer primarily through serine hydroxymethyltransferase 2 (SHMT2), supplying raw materials for purine and pyrimidine biosynthesis as well as DNA methylation. This mechanism is conservatively expressed in colorectal cancer, bladder cancer, and acute myeloid leukemia, constituting an essential metabolic foundation for the rapid proliferation of tumor cells. For drug resistance regulation, metabolic reprogramming mediated by SSP activation represents a common mechanism underlying chemoresistance and targeted therapy resistance across pan-cancer. For example, the resistance of colorectal cancer to 5-fluorouracil (5-FU), melanoma to MEK inhibitors, and hepatocellular carcinoma to lenvatinib is all associated with enhanced SSP activity, which promotes cell survival by improving metabolic plasticity.

#### 4.3.2 Cancer-type-specific adaptive strategies

Driven by distinct microenvironmental characteristics and driver mutation profiles, different cancer types evolve specific adaptive strategies for SSP activation, reflecting the remarkable metabolic plasticity of tumors.

##### 4.3.2.1 Driver mutation-dependent pattern

The driver mutation-dependent pattern is mainly observed in pancreatic cancer (harboring Kras mutations), bladder cancer (harboring FGFR3 mutations), and acute myeloid leukemia (harboring FLT3-ITD mutations). In these cancer types, specific driver mutations directly upregulate the expression of key SSP enzymes, tightly coupling SSP activity with mutational status and forming a malignant proliferation loop characterized by “mutation-metabolism” crosstalk. For example, Kras mutations reduce the dependence of pancreatic cancer cells on extracellular serine, enabling metabolic self-sufficiency.

##### 4.3.2.2 Microenvironmental stress-adaptive pattern

The microenvironmental stress-adaptive pattern is represented by glioblastoma, pancreatic cancer, and bladder cancer. In response to serine deprivation in the brain and nutrient deprivation in the tumor microenvironment, these tumors maintain metabolic demands through full-pathway SSP activation and microenvironmental remodeling. For instance, glioblastoma enhances stem cell self-renewal via MYC-mediated SSP activation to adapt to the nutrient-poor cerebral microenvironment (64). Pancreatic cancer promotes tumor innervation by secreting nerve growth factor (NGF), compensating for insufficient exogenous nutrients by utilizing serine released from nerve axons (73), thereby establishing an adaptive loop of “metabolism-microenvironmental remodeling”. In bladder cancer, following FGFR3 mutation-driven SSP activation, increased serine synthesis activates the PI3K/Akt pathway in macrophages, inducing the formation of immunosuppressive macrophages and constructing an “immune-desert” microenvironment to facilitate immune escape (42). This mechanism highlights the cancer-type-specific role of the SSP in tumor immune regulation.

##### 4.3.2.3 Epigenetic dysregulation subtype

Hepatocellular carcinoma and breast cancer are typical examples of the epigenetic dysregulation subtype. In hepatocellular carcinoma, PRMT1 catalyzes the methylation of PHGDH at residue R236 to enhance its enzymatic activity (29), whereas FBXO7-mediated degradation of PRMT1 reverses SSP hyperactivation (31). In breast cancer, hypermethylation of the PSAT1 promoter is associated with T-cell dysfunction (44). In AML, the m6A-IGF2BP3 axis stabilizes the transcripts of SSP-related genes and sustains the maintenance of leukemia stem cells (93).

The tumor classification based on SSP activation patterns reveals that the conserved mechanisms of SSP, including redox homeostasis maintenance, one-carbon metabolism and drug resistance regulation, can serve as universal targets for pan-cancer therapeutic interventions. In contrast, the cancer-specific adaptive strategies, namely driver mutation dependence, microenvironmental stress adaptation and epigenetic dysregulation, necessitate the design of tailored therapeutic regimens. For instance, for the driver mutation-dependent subtype, a combined strategy of targeted therapy and epigenetic regulation should be adopted in accordance with molecular subtypes, such as the FGFR3-mutant subtype in bladder cancer. For the microenvironmental stress-adaptive subtype, the combination of SSP-targeted therapy and microenvironmental remodeling pathways is feasible, for example, by simultaneously targeting the SSP and the NGF-mediated tumor innervation pathway in pancreatic cancer. For the epigenetic dysregulation subtype, the priority is to use epigenetic drugs in combination with SSP inhibitors, such as m6A inhibitors plus PHGDH inhibitors, to abrogate the aberrant activation of genes encoding key SSP metabolic enzymes at the source.

In summary, through pan-cancer analysis using the TIMER database combined with mechanistic integration, this review demonstrates that the activation patterns of the serine synthesis pathway (SSP) in malignant tumors are not randomly distributed, but follow three distinct molecular subtypes: driver mutation-dependent, microenvironmental stress-adaptive, and epigenetic dysregulation subtypes. Tumors with concurrent high expression of all three SSP enzymes exhibit full-pathway activation of the SSP. These tumors maintain redox homeostasis and DNA repair via metabolic self-sufficiency. We hypothesize that these tumors may be sensitive to monotherapy with PHGDH inhibitors. In contrast, cancers with high expression of only one or two enzymes display metabolic flux redirection or environmental dependence, requiring combination strategies targeting transporter bypass pathways or microenvironmental remodeling cascades. Cross-cancer comparisons reveal that although the SSP serves conserved functions in sustaining one-carbon unit supply and mediating therapeutic resistance across cancer types, the remarkable heterogeneity of its upstream regulatory mechanisms—including Kras/FGFR3 mutations, MYC-mediated stemness maintenance, and m6A epigenetic modifications—dictates that therapeutic strategies must be precisely tailored. For driver mutation-dependent tumors, targeted drugs should be combined to interrupt the rigid coupling between oncogenic mutations and metabolic activation. For the microenvironmental stress-adaptive subtype, metabolic intervention should be integrated with microenvironmental modulation. For tumors of the epigenetic dysregulation subtype, synergistic therapy combining epigenetic agents with SSP inhibitors represents the optimal approach.

## 5 Multilevel regulatory mechanisms of the *de novo* serine synthesis pathway in malignant tumors

### 5.1 Transcription factor regulation

Multiple key transcription factors have been verified to directly regulate the expression of core enzymes in the SSP. c-Myc can directly bind to the promoter regions of PHGDH and PSAT1 and promote their transcription, a mechanism that has been validated in breast cancer and glioblastoma (64, 98, 99). HIF-1α upregulates PHGDH expression in the hypoxic tumor microenvironment, sustaining tumor cell survival under hypoxic conditions by enhancing serine synthesis (100).

In non-small cell lung cancer, NRF2 upregulates the expression of PHGDH, PSAT1 and SHMT2 by activating ATF4, thereby driving SSP activation, which is associated with poor clinical prognosis (101). Under serine deprivation, ATF4 is induced and activates the transcription of SSP-related genes. ATF3 enhances the stability of ATF4 by forming a complex with it, and directly binds to the promoter or enhancer regions of PHGDH, PSAT1 and PSPH to recruit the p300 coactivator, thereby synergistically amplifying the transcriptional activation of the SSP (102). In endometrial cancer, the oncogenic factor SOX12 has been shown to directly bind to the PHGDH promoter and activate its transcription, thus enhancing SSP activity and promoting malignant tumor progression (103). As a tumor suppressor, YY2 negatively regulates *de novo* serine synthesis by binding to the PHGDH promoter and repressing its transcriptional activity, thereby inhibiting tumorigenesis (104).

### 5.2 Epigenetic regulation

mRNA modifications are also implicated in the regulation of the *de novo* serine synthesis pathway. In colorectal cancer, NSUN2 enhances the stability of PHGDH mRNA through m5C methylation, thereby activating the SSP, increasing the intracellular NADH/NAD^+^ and NADPH/NADP^+^ ratios, reducing ROS levels and apoptosis, and promoting tumor cell survival and proliferation (105). In acute myeloid leukemia, the m6A methyltransferase METTL3 and the reader protein IGF2BP3 maintain high-level serine synthesis by recognizing and stabilizing the mRNAs of SSP-related genes including ATF4, PHGDH and PSAT1, meeting the high metabolic demands of cancer cells, particularly leukemia stem/initiating cells (93).

### 5.3 Post-translational modification regulation of enzyme activity

**Figure 3:**
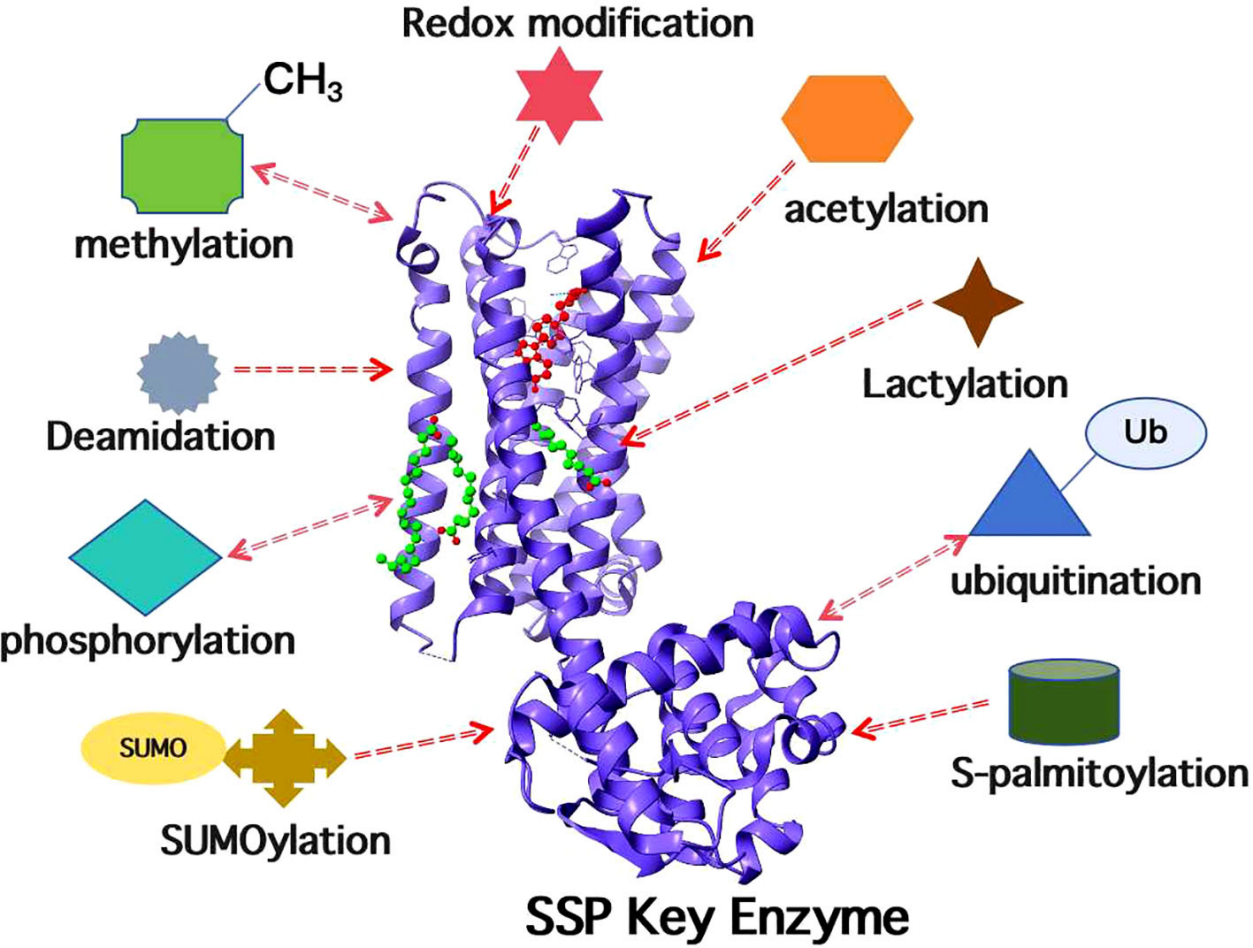
Schematic diagram of post-translational modifications regulating the activity of key SSP enzymes. The activity and stability of core enzymes in the SSP, including PHGDH, PSAT1 and PSPH, are precisely regulated through diverse post-translational modifications.

The activity of metabolic enzymes involved in the *de novo* serine synthesis pathway is precisely modulated by post-translational modifications such as methylation, ubiquitination and deacetylation (**Figure 3**), and such regulatory patterns exhibit cancer-type specificity. In hepatocellular carcinoma, FBXO7 indirectly suppresses the PRMT1-mediated methylation and activation of PHGDH by promoting the ubiquitination and degradation of PRMT1, thereby inhibiting serine synthesis and tumor growth (31).

Multiple E3 ubiquitin ligases influence SSP activity by regulating the stability of PHGDH, the rate-limiting enzyme of the pathway (106, 107). As a tumor suppressor, Parkin directly binds to PHGDH and catalyzes its ubiquitination at lysine 330, leading to proteasomal degradation. Downregulation of Parkin in various cancers results in PHGDH protein accumulation and SSP activation, thus driving tumorigenesis (108, 109).

Argininosuccinate synthase 1 (ASS1) functions as a tumor suppressor in triple-negative breast cancer (TNBC). ASS1 directly interacts with PHGDH and promotes its ubiquitination and degradation. Elevated PHGDH restricts serine biosynthesis, while ASS1 knockdown partially rescues TNBC progression under serine deprivation (70). In colorectal cancer, the p185 protein encoded by the circular RNA circMYBL2 competitively binds to the deubiquitinase UCHL3, preventing UCHL3 from deubiquitinating PHGDH, which consequently leads to PHGDH degradation and suppresses serine synthesis and tumor progression (110).

HDAC7 has been identified as the deacetylase of PSAT1 and catalyzes PSAT1 deacetylation at lysine 51. HDAC7-mediated deacetylation stabilizes PSAT1, thereby affecting serine metabolism and the sensitivity of lung adenocarcinoma (LUAD) cells to the chemotherapeutic agent cisplatin (111).

### 5.4 Cross-regulation by signaling pathways

The *de novo* serine synthesis pathway forms an interactive regulatory network with oncogenic signaling pathways including Akt, mTOR and EGF-ERK. In triple-negative breast cancer (TNBC), PSAT1 drives tumor metastasis by activating the AKT/SP1/ITGA2 axis (112). The mTOR pathway regulates the activity of PSPH via phosphorylation, participating in the dynamic modulation of serine synthesis and tumor growth across pan-cancer. For instance, upregulation of the SSP is driven by the AMP-activated protein kinase–mTORC1 axis, leading to elevated S-adenosylmethionine (SAM) production and increased expression of DNA methyltransferases, thereby promoting oncogenic transformation of primary pancreatic ductal epithelial cells (9).

In hepatocellular carcinoma (HCC), USP10 activates liver kinase B1 (LKB1) through deubiquitination. The LKB1/mTOR/ATF4 axis acts as an upstream regulatory module governing the SSP by modulating the transcription of SSP-related enzymes, thereby influencing cell proliferation (113). In cholangiocarcinoma, combined BET degradation and mTOR inhibition synergistically reduce acetylation of PSAT1-associated H3K27, inducing PSAT1 downregulation and subsequent dysfunction of the serine-glycine-one-carbon (SGOC) pathway (114).

In colorectal cancer, the EGF-MEK-ERK signaling pathway mediates phosphorylation of ILF3, protecting it from degradation by the E3 ubiquitin ligase SPOP. High levels of ILF3 promote SSP flux by enhancing the mRNA stability of multiple genes involved in serine-glycine-one-carbon metabolism, thereby driving tumor growth (115). In glioblastoma (GBM), AMPK-HIF-1α signaling reinforces glucose-derived *de novo* serine synthesis to facilitate tumor growth (100).

Taken together, the *de novo* serine synthesis pathway (SSP) in malignant tumors is subjected to multilevel regulation encompassing transcription, epigenetic modification and post-translational modification. These regulatory layers coordinately target key enzymes including PHGDH and PSAT1, adapting their expression, stability and activity to meet the metabolic demands of tumor cells, and the regulatory patterns exhibit obvious cancer-type specificity. Transcription factors exert both positive and negative control over SSP-related gene transcription. Epigenetic modifications maintain the stability of target mRNAs through various mRNA methylation events. Post-translational modifications, such as ubiquitination, precisely modulate enzymatic activity. Collectively, these regulatory mechanisms provide critical insights and potential targets for the study of tumor metabolic reprogramming and targeted therapy development.

## 6 Pan-cancer therapeutic potential of targeting the *de novo* serine synthesis pathway

### 6.1 Therapeutic strategies targeting the SSP

Given the pivotal role of the SSP in carcinogenesis, targeting this pathway has emerged as a promising therapeutic strategy. Current research focuses on three major approaches: dietary intervention, development of key enzyme inhibitors, and combination therapy. The synergistic application of these strategies is expected to overcome the limitations of single-agent treatment (**Table 1**).

**Table 1:** Development of therapeutic strategies targeting the *de novo* serine synthesis pathway.

Strategy type	Specific approach	Research phase	Advantages	References
Dietary Intervention	Restriction of serine/glycine intake	Clinical Trial	Enhances the efficacy of chemotherapy/radiotherapy	(116, 117)
Enzyme Inhibitor	PHGDH inhibitor CBR-5884	Preclinical	Reverses drug resistance when combined with tyrosine kinase inhibitors (TKIs)	(33, 63, 118)
	PHGDH inhibitor NCT-503	Preclinical	Overcomes drug resistance	(119)
	PHGDH inhibitor PKUMDL-WQ-2201	Preclinical	Inhibits breast cancer progression	(120)
Methylation Blockade	PHGDH-R236 methylation inhibitor STM2457	Preclinical	Effectively suppresses hepatocellular carcinoma (HCC) growth in PDX models	(61)
Ubiquitination-Mediated Degradation	Ubiquitination and proteasomal degradation of PHGDH-PSAT1 via ARV825	Preclinical	Exerts synergistic antitumor effects with mTOR pathway inhibitors	(114)
Combination with Immunotherapy	PHGDH inhibitor combined with chimeric antigen receptor T (CAR-T) cell therapy	Preclinical	Restores serine levels in the tumor microenvironment (TME) and enhances T cell function	(47)

#### 6.1.1 Dietary intervention

Dietary restriction of serine and glycine intake can suppress the growth of serine-dependent tumors (9, 116, 117). The core mechanism lies in cutting off the supply of metabolic precursors for tumor cells reliant on exogenous serine, while simultaneously perturbing the one-carbon metabolic cycle, thereby inhibiting nucleotide biosynthesis and cell proliferation. The efficacy of this strategy has been validated in multiple tumor models. For instance, in mouse models of colorectal cancer and melanoma, serine-restricted diets significantly delay tumor growth and enhance the cytotoxic effects of chemotherapeutic agents (55, 121). Serine restriction also reprograms the tumor immune microenvironment from an immunosuppressive state to an immune-permissive state, thereby constraining tumor progression (122).

Notably, the efficacy of dietary restriction is tumor-specific: it only exerts potent effects on tumors highly dependent on the SSP, while showing limited efficacy in tumors with low SSP dependence or those capable of compensatory activation of the endogenous SSP (102). Furthermore, long-term dietary restriction may cause systemic metabolic disorders (123), necessitating individualized regimens tailored to tumor type and patient physical condition. Current clinical trials are exploring the feasibility of combining this approach with conventional therapies.

#### 6.1.2 Inhibitors targeting key SSP enzymes

Developing inhibitors against key enzymes in the *de novo* serine synthesis pathway has become an important direction for pan-cancer therapy. The PHGDH inhibitor NCT-503 reverses enzalutamide resistance in prostate cancer (34) and effectively suppresses tumor growth in hepatocellular carcinoma. In contrast, targeting peptides against PHGDH methylation modifications (e.g., non-methylated cell-penetrating peptides) can competitively inhibit the binding of PRMT1 to PHGDH, exhibiting favorable antitumor effects with no obvious toxic side effects in hepatocellular carcinoma patient-derived xenograft (PDX) models (29).

In preclinical models, PHGDH inhibitors demonstrate therapeutic efficacy against IDH2-driven breast cancer (71), endometrial cancer (124), pancreatic cancer (3), and cancers with Parkin deficiency (109). Given the pharmacological limitations of existing PHGDH inhibitors, such as poor aqueous solubility and insufficient target specificity, nano-delivery systems have significantly improved tumor accumulation and therapeutic index through passive and active targeting strategies. For example, acid-responsive NCT-503@Cu-HMPB nanoparticles enable tumor microenvironment-specific drug release, effectively inhibiting tumor growth while reducing systemic toxicity (125).

Small-molecule inhibitors of PSAT1 identified via computational chemistry screening represent potential therapeutic candidates (126), although their efficacy and safety across pan-cancer require further validation. At present, PSPH inhibitors remain in the preclinical research stage, with no candidate drugs advancing to advanced clinical trials.

#### 6.1.3 Combination therapy

Studies have shown that combining PHGDH inhibitors with serine/glycine-restricted diets yields synergistic antitumor effects. This combination more effectively disrupts one-carbon metabolism and interferes with protective stress responses (such as the ATF4-mediated stress response), thereby overcoming drug resistance that may arise from single-agent or single-dietary interventions (127). In colorectal cancer, the combination of one-carbon metabolism inhibitors with anti-EGFR antibodies (e.g., cetuximab) has been proven to effectively suppress the growth of tumors with high ERK-ILF3 expression (115).

In hepatocellular carcinoma, the combination of m6A inhibitors with sorafenib or lenvatinib reverses drug resistance (61). Inhibition of PHGDH enhances the sensitivity of glioblastoma to CAR-T cell therapy (47), while the methylation status of PSAT1 can serve as a predictive biomarker for immunotherapy response in breast cancer (44), providing a basis for precision therapy.

### 6.2 The SSP as a key metabolic pathway mediating resistance to conventional chemotherapy and targeted therapy

The SSP serves as a pivotal metabolic pathway that mediates tumor resistance to conventional chemotherapeutic agents and targeted therapeutics. Drug-resistant tumors can upregulate the activity of key SSP enzymes to enhance serine synthesis or counteract reactive oxygen species (ROS) accumulation. Therefore, targeting the SSP holds promise for reversing drug resistance and represents a novel strategy to overcome therapeutic resistance. Based on the type of therapeutic agents, SSP-mediated drug resistance can be categorized into chemotherapy resistance and targeted therapy resistance, with distinct regulatory logics and intervention potentials.

#### 6.2.1 Chemotherapy resistance

##### 6.2.1.1 Doxorubicin resistance

Triple-negative breast cancer cells exposed to doxorubicin undergo metabolic reprogramming, leading to elevated PHGDH-dependent serine synthesis. Serine is subsequently converted into glutathione (GSH) to counteract doxorubicin-induced ROS production. Consequently, PHGDH inhibition enhances cellular sensitivity to doxorubicin (101). PHGDH knockdown in estrogen receptor-positive (ER^+^) breast cancer cells exposed to cytotoxic chemotherapy (carboplatin or doxorubicin) results in increased mitochondrial ROS and prevents chemotherapy-induced enrichment of cancer stem cells (CSCs) (128). Thus, PHGDH may represent a novel therapeutic target for reversing recurrence and resistance to tamoxifen in ER^+^ breast cancer (129).

##### 6.2.1.2 5-Fluorouracil resistance

Metabolic plasticity of serine metabolism constitutes one of the critical metabolic pathways underlying 5-fluorouracil (5-FU) resistance in colorectal cancer. Enhanced SSP activity promotes colorectal cancer progression and contributes to 5-FU resistance. Accordingly, serine deprivation or SSP blockade restores the sensitivity of colorectal cancer cells to 5-FU (55).

#### 6.2.2 Targeted therapy resistance

##### 6.2.2.1 BRAF inhibitor resistance

Proteomic analysis of acquired-resistance cell lines revealed altered expression of serine biosynthetic enzymes (PHGDH, PSPH, PSAT1) in drug-resistant cells. Knockdown of PHGDH via siRNA resensitizes previously resistant melanoma cells to vemurafenib, a BRAF inhibitor (3). Similarly, inhibition of the folate cycle downstream of serine metabolism using methotrexate also yields comparable sensitization effects (130). Moreover, pretreatment with gemcitabine, a DNA-damaging agent, significantly enhances the cytotoxicity of BRAF inhibitors against resistant melanoma, pancreatic cancer and non-small cell lung cancer cells (130).

##### 6.2.2.2 Sorafenib resistance

In hepatocellular carcinoma, high PHGDH expression acts as a major driver of sorafenib resistance. Inhibition of PHGDH reduces the production of α-ketoglutarate (α-KG), serine and NADPH, and induces cell death by elevating ROS levels (131).

##### 6.2.2.3 EGFR-TKI resistance

Halofuginone (HF), a natural product, exhibits potential in reversing epidermal growth factor receptor tyrosine kinase inhibitor (EGFR-TKI) resistance. HF downregulates PSAT1 expression by promoting SP1 protein degradation, thereby interfering with *de novo* serine/glycine synthesis and ultimately inducing death in drug-resistant cells. Preclinical studies confirm that combined treatment with HF and an EGFR-TKI exerts synergistic cytotoxicity compared with EGFR-TKI monotherapy, highlighting promising application prospects, especially in overcoming resistance to third-generation EGFR-TKIs (132).

### 6.3 Critical assessment and challenges of SSP-targeted therapy

#### 6.3.1 Target specificity and toxicity evaluation in normal tissues

The SSP is not only aberrantly activated in tumor cells but also participates in fundamental metabolism in normal tissues, including the nervous system, immune cells and the liver. Systemic inhibition of the SSP may induce potential toxicities and narrow the therapeutic window. In the nervous system, serine serves as a precursor for neurotransmitter biosynthesis, and the central nervous system is highly dependent on exogenous serine supply. Long-term dietary restriction or PHGDH inhibition may lead to neurological disorders, such as cognitive impairment and abnormal motor coordination (123, 133), as well as hepatic dysfunction (134).

In immune cells, the SSP is indispensable for maintaining the function of CD8^+^ T cells and macrophages. Excessive SSP suppression may impair the host’s antitumor immune response and inadvertently promote tumor progression (43, 47). Regarding the therapeutic window: dietary restriction confers relatively mild toxicity, and the risk can be reduced through individualized dose adjustment, yet its antitumor efficacy is limited. Specific PHGDH inhibitors exhibit lower toxicity and a wider therapeutic window by targeting tumor-specific modification sites (135). In contrast, broad-spectrum SSP inhibitors carry a higher risk of toxicity due to interference with normal tissue metabolism, necessitating optimization via targeted delivery technologies (136, 137).

Although most SSP inhibitors evaluated in preclinical studies have not shown severe systemic toxicity, close monitoring of neurological, immune and hepatic functions is essential during clinical translation.

#### 6.3.2 Distinguishing causality from correlation in SSP-mediated drug resistance

A strong correlation between SSP upregulation and tumor drug resistance has been widely documented; however, the strength of causal evidence varies across different resistance models.In BRAF inhibitor resistance, PHGDH knockdown reverses resistance, and exogenous serine supplementation rescues the resistant phenotype. In sorafenib resistance, high PHGDH expression directly drives resistance and can be directly reversed by pharmacological inhibition. In 5-FU resistance, serine deprivation restores chemosensitivity, and metabolite supplementation recapitulates resistance. These studies, using genetic manipulation and metabolite rescue experiments, confirm that the SSP acts as a driver of drug resistance.

By contrast, in EGFR-TKI resistance, only a correlation between PSAT1 downregulation and reversed resistance has been established. The absence of metabolite rescue experiments makes it impossible to rule out the possibility that SSP upregulation represents an adaptive response following resistance acquisition (126). Furthermore, SSP upregulation in some studies may be associated with global metabolic reprogramming in tumor cells, rather than directly driving resistance (138, 139). More rigorous mechanistic validation, such as conditional knockout models and metabolic flux analysis, is required to distinguish driver roles from concomitant responses.

#### 6.3.3 Core challenges and future directions

Several critical challenges remain to be addressed for the clinical translation of SSP-targeted therapy. Functional heterogeneity: The SSP exerts opposing functions in tumor cells versus tumor-associated cells, such as M2-type macrophages and CD8^+^ T cells. For example, PHGDH promotes immune escape in tumor cells but suppresses antitumor T-cell immunity in endothelial cells, revealing cell-type-specific functional dichotomy of the SSP (47). A key goal is to precisely target the SSP in tumor cells without compromising immune cell function.

Microenvironmental dynamics: Biophysical cues including extracellular matrix stiffness modulate serine synthesis by regulating glycolysis, but the underlying molecular mechanisms remain poorly defined, limiting the development of combined strategies targeting both metabolism and the tumor microenvironment.

Lack of pan-cancer systematic analysis: Most current studies focus on single cancer types. The heterogeneity of SSP expression profiles, functional outputs and regulatory networks across cancer types has not been fully elucidated, hindering the design of pan-cancer therapeutic strategies.

Future research should employ multi-omics integration to decipher pan-cancer regulatory patterns of the SSP, develop cell-specific targeting agents, and combine metabolic intervention with microenvironmental modulation and rational combination therapies. These strategies will help overcome bottlenecks of drug resistance and systemic toxicity, ultimately accelerating the clinical translation of SSP-targeted therapies.

## 7 Summary and outlook

The *de novo* serine synthesis pathway (SSP) represents a highly dynamic and precisely regulated core metabolic node across pan-cancer. It not only supports the bioenergetic and biosynthetic demands of tumors, but also profoundly influences metastatic potential and therapeutic response. Its regulatory network encompasses multiple layers, including post-translational modification, transcriptional regulation and crosstalk with signaling pathways, with marked heterogeneity in regulatory patterns across distinct cancer types. Future research will continue to explore the cancer-specific regulatory mechanisms of the SSP in diverse oncological contexts, and promote the development of precision therapeutic strategies targeting this pathway. For instance, biomarkers such as high PHGDH expression, IDH2 status and Parkin deficiency can be utilized to identify patient populations most likely to benefit from SSP-targeted therapy. The combination of SSP targeting with radiotherapy, chemotherapy and immunotherapy is expected to become an effective approach to overcome tumor heterogeneity and therapeutic resistance.
